# Immediate Loading and Implant Placement With Bone Grafting in Severely Proclined Anterior Mobile Teeth in the Esthetic Zone: A Report of an Intriguing Case

**DOI:** 10.7759/cureus.71541

**Published:** 2024-10-15

**Authors:** Rimmy Takvani, Aakash Takvani, Arwa Pethapur, Shashank Kaushik

**Affiliations:** 1 Prosthodontics, Goenka Research Institute of Dental Science, Gandhinagar, IND; 2 Prosthodontics, Takvani Dental and Implants, Jamnagar, IND; 3 Dentistry, Government Dental College and Hospital, Jamnagar, IND; 4 Dentistry, Takvani Dental and Implants, Jamnagar, IND

**Keywords:** bone grafting, early loading, esthetics, esthetic smile designing, failed orthodontics, immediate loading, immediate placement, implants, mobile teeth

## Abstract

Achieving success in dental implant therapy within the esthetic zone involves not only ensuring that the implant properly integrates with the surrounding bone but also achieving a visually pleasing outcome. It is challenging to create a seamless and balanced appearance of the gums around the implant, in harmony with the natural teeth nearby. To replace a missing tooth in the esthetic zone, immediate implant placement and provisionalization, a meticulously researched and dependable method, have received recognition as a predictable technique demanding fewer surgical procedures. This case report demonstrates how to replace failing upper central and lateral incisors while maintaining the gum esthetics by using immediate implant placement and provisionalization. In order to reduce trauma to both soft and hard tissues, the upper right and left central and lateral incisors were extracted without elevating the gum flap. Subsequently, a dental implant was promptly inserted using a surgical guide with bone graft and furnished with a provisional crown that did not contact the opposing teeth. Throughout the healing period, no notable negative effects were observed in both clinical assessments and X-rays. This proposed treatment approach offered the patient immediate improvements in esthetics, functionality, and comfort without encountering any complications.

## Introduction

Dental implants have emerged as an acceptable and effective treatment option for people who have lost all or some of their teeth. The idea of osseointegration was first proposed by Dr. Brånemark in 1968, which was a major turning point for implant dentistry. The efficacy and dependability of restorations supported by dental implants have now been evaluated by a great deal of clinical research backed by extended follow-up investigations [1] in order to prevent any functional loading and subsequently discovered during a second minor operation. It has been shown that two-stage implants placed using a one-stage process are just as effective as two-stage implants implanted during a two-stage technique. They are often inserted in two stages, with the first step involving their total submersion beneath the mucosal tissue, which allows for a predictable healing phase [2]. Moreover, equivalent clinical and radiographic outcomes have been observed during the healing phase in a prospective clinical investigation comparing one-stage implants with two-stage implants implanted using a one-stage method [3]. Because of this, more and more implants are now inserted during implant surgery in a single step, at where the implant fixture and healing abutment are connected.

In the past, it was customary to wait three months following tooth extraction to place a dental implant since this allowed the hard and soft tissues to heal. For those with partial tooth loss, the tried-and-true method of implanting a prosthesis into a healed socket and then restoring it has shown to be an extremely effective course of treatment [4].

When primary implant stability is attained and the provisional restoration can be modified to meet centric and eccentric contacts, immediate implant placement and provisionalization emerge as a viable treatment option for replacing a compromised tooth in the esthetic zone.

## Case presentation

**Figure 1:**
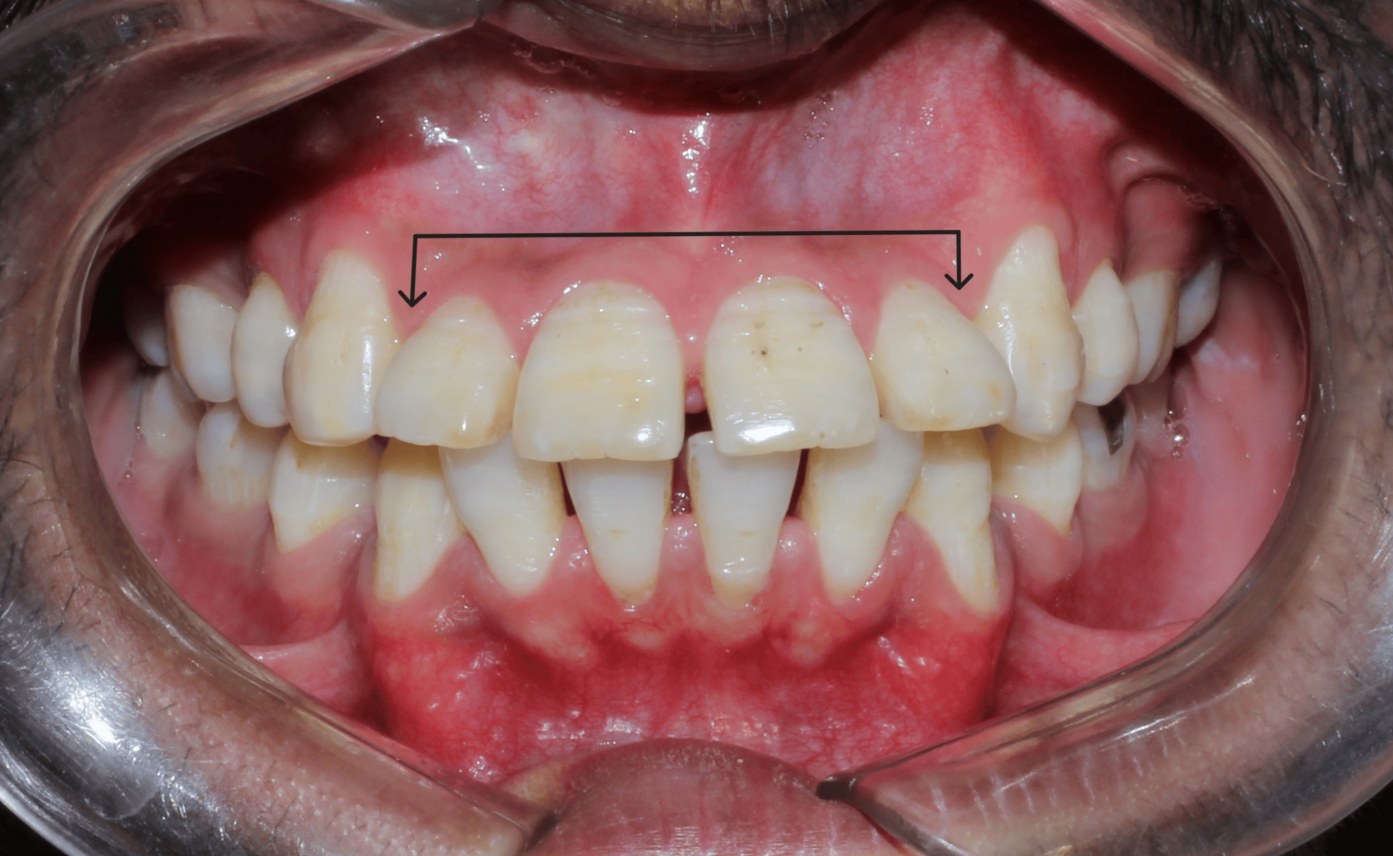
Proclined upper anteriors.

**Figure 2:**
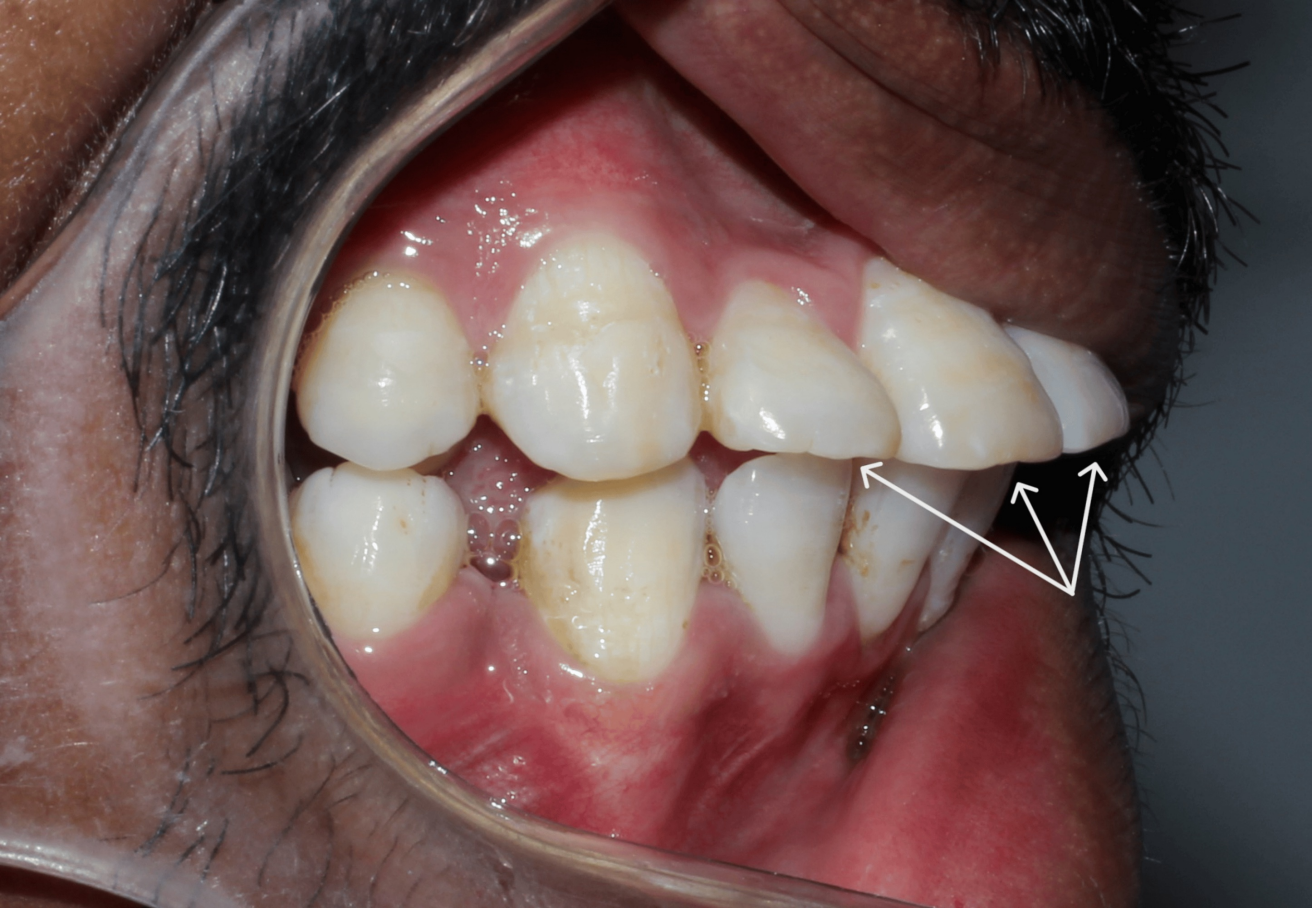
Lateral view showing flared upper anteriors past failed orthodontic treatment.

**Figure 3:**
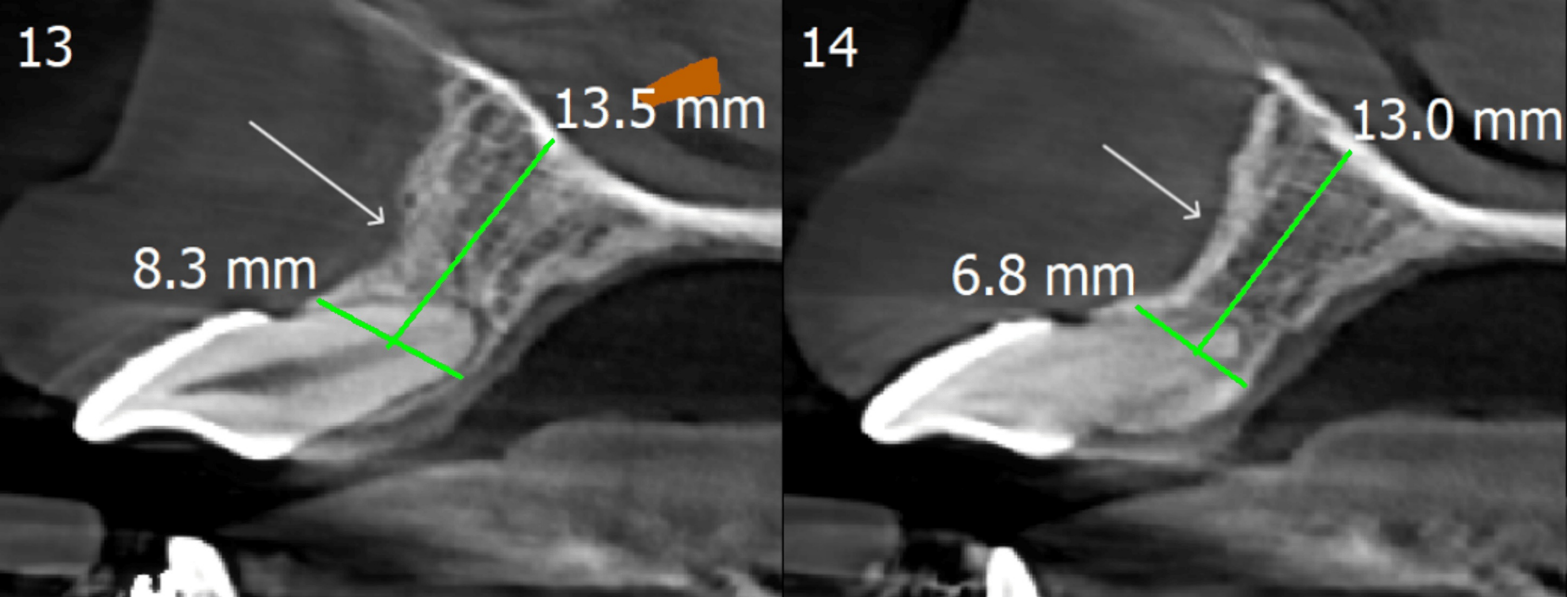
CBCT slide showing labioversion of incisors with compromised buccal plate of the bone. CBCT: cone-beam computed tomography

A 22-year-old male patient visited Takvani Dental and Implants for the correction of his proclined maxillary anteriors. He gave a verbal history of having undergone orthodontic treatment for about two years at a different clinic but was not satisfied with the results as shown in Figure 1 and Figure 2. Since the previous treatment was done at another clinic, the patient reported not holding any previous records, which include casts as well as radiographs. He had no significant medical history, had no history of smoking, and was classified as American Society of Anesthesiologists (ASA) Physical Status Classification System-I. A comprehensive periodontal examination revealed no periodontal pocket depth beyond 3-4 mm. His upper anteriors were grade II mobile (according to the classification presented by Preston D. Miller) due to excessive bone loss (as shown in Figure 3) as a result of extensive orthodontic treatment. There was no pain on percussion or palpation in teeth from 12 to 22. After clinical and radiographic pre-operative analysis (cone-beam computed tomography (CBCT) shown in Figure 3) to assess the patient's risk profile for immediate implant placement with provisionalization after tooth extraction was recommended, the patient accepted the treatment plan. The opinion of the orthodontist on braces or aligners was taken, and it was reported that the upper arch will have a poor prognosis owing to loss of bone and mobility, so a lateral cephalometric radiograph was not made.

**Figure 4:**
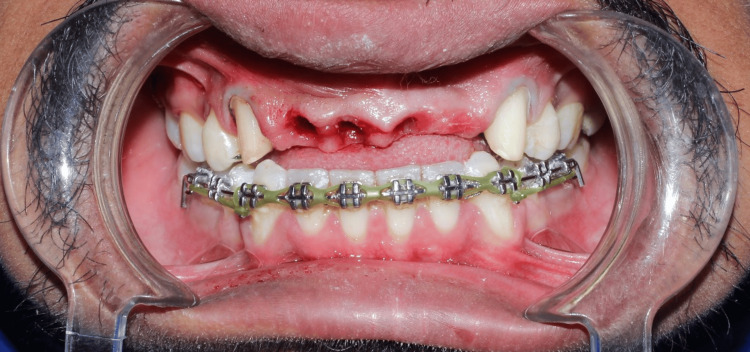
Extraction of upper central and lateral incisors is done.

**Figure 5:**
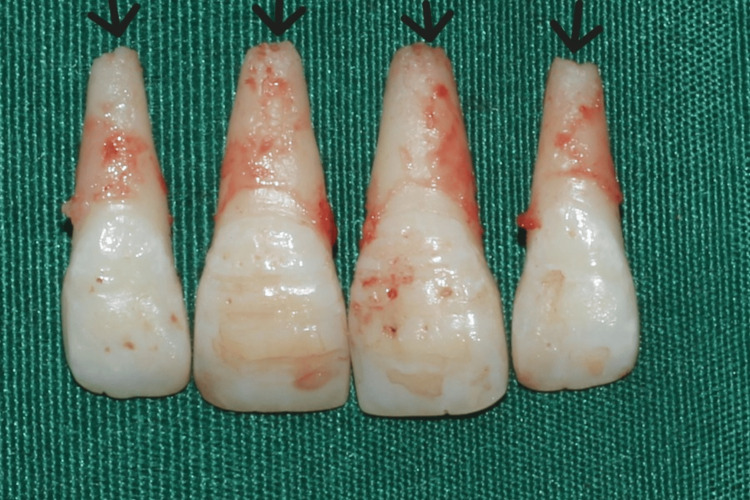
Extracted teeth showing signs of root resorption may be suggestive of excessive forces.

**Figure 6:**
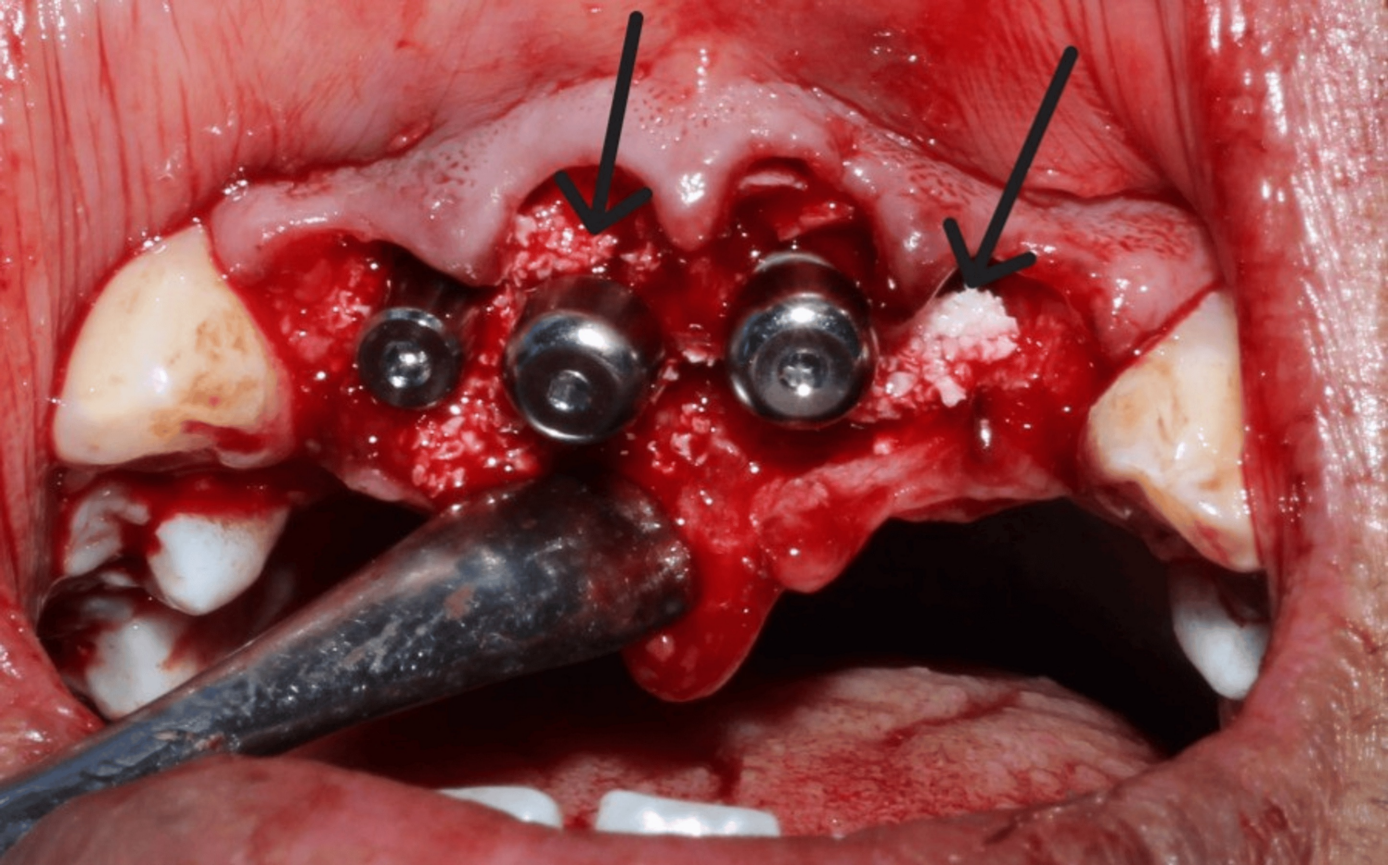
Palatal placement of implant along with GBR using Bio-Oss and Periocon (Bio-Oss, Geistlich, Wolhusen, Switzerland). GBR: guided bone regeneration

The patient received oral prophylaxis and was prescribed a combination of amoxiclav and clavulanic acid 625 mg three times a day for five days as antibiotic medicine for surgical prophylaxis starting from the day before the procedure to increase the implant's success rate [5]. A 12C blade was used to make a sulcular incision around teeth 12-22 under local anesthetic in order to sever the connective tissue fibers above the bone. After that, the teeth were extracted without raising the flap using extraction forceps with regulated rotating force. The buccal plate in particular was already compromised (as shown in Figure 3) due to failed orthodontic treatment; still, we tried and succeeded to prevent fracture of the socket walls in order to preserve the gingival and bone architecture. Before the osteotomy, curettage of the extraction socket was done, and sterile cold saline irrigation was used. The integrity of the socket walls and gingiva and alveolar crest were both confirmed using a periodontal probe. CBCT analysis interpreted that there was a lack of buccal plate of the bone; hence, grafting was required. To improve graft uptake, a portion of the buccal plate has been used as an autograft in conjunction with Bio-Oss (Geistlich, Wolhusen, Switzerland) xenograft (Figures 4-6). The palatal wall of the extraction socket was broken up with a sharp precision drill (OSSTEM IMPLANT, Seoul, South Korea), which served as a guide for the initial osteotomy preparation. A periapical radiograph was obtained using a twist drill. The rule of restorative-driven three-dimensional placement was immediately followed when placing two Myriad implants of size 3.8×13 mm, with respect to 22 and 21, and one Bioline implant of size 3.75×10 mm, with respect to 12, and then grafting followed by sutures taken by 5-0 non-absorbable, 3/8 circle reverse cutting needle [6].

The implant's primary stability was validated with a torque resistance of 35-40 Ncm. Demineralized freeze-dried bone allograft (Bio-Oss) was placed into the space between the labial bony wall and the implant. A provisional bridge with an ovate pontic design was fabricated with indirect method to protect the blood clot and bone graft while maintaining the soft tissue's emerging profile, and this temporary bridge was cemented with a glass ionomer cement (GIC) during the healing phase. The temporary bridge's occlusion was modified to create disclusion during maximum intercuspation. The patient was advised not to apply any pressure to the temporary bridge while it was healing. Following surgery, antibiotics were continued, and analgesics were added to control infection and discomfort. For two weeks, mouth rinses containing 2% chlorhexidine gluconate were recommended twice a day. The patient was counseled to refrain from using force on their anterior teeth and to follow a soft diet.

**Figure 7:**
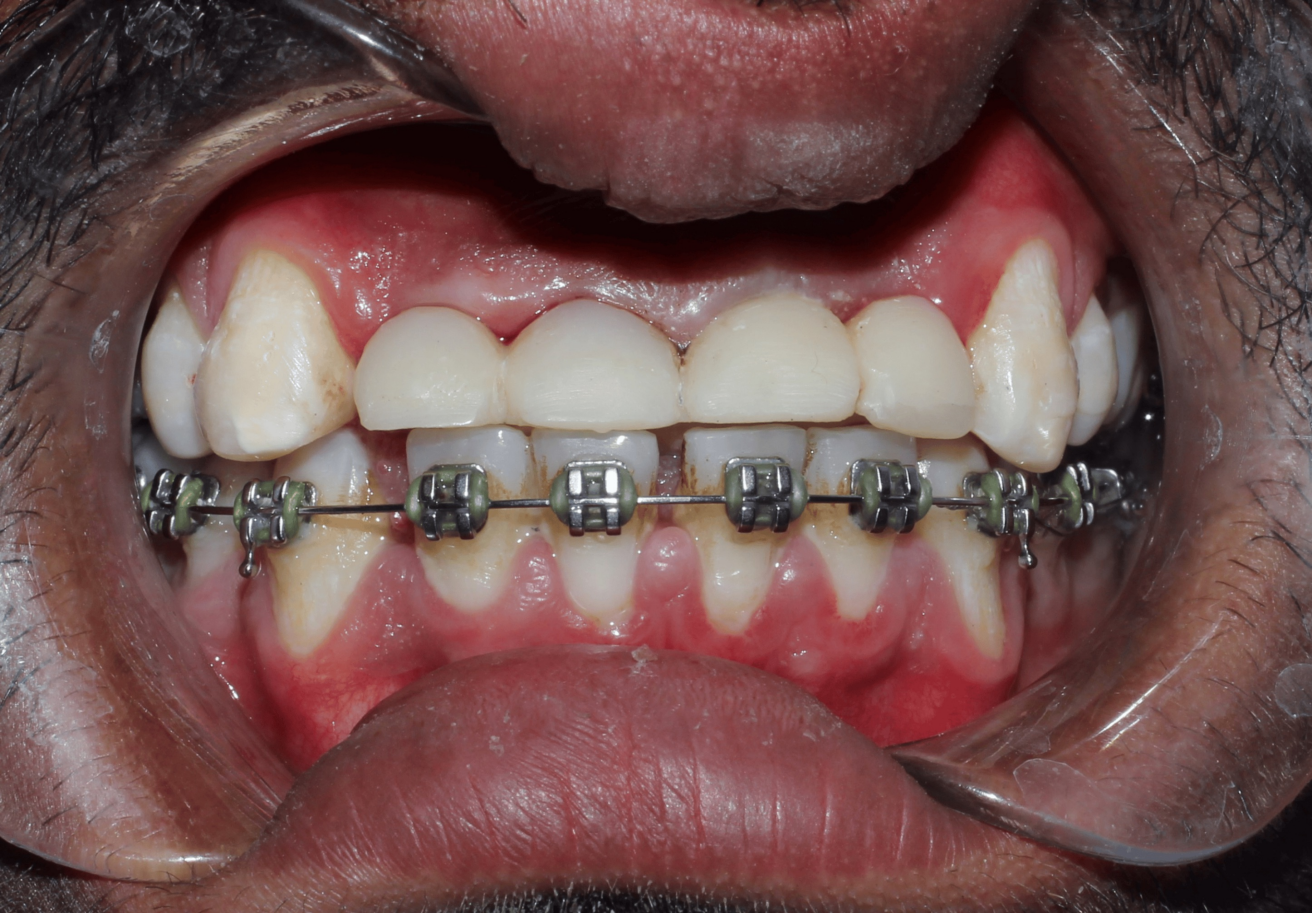
Frontal view showing temporisation of teeth with disclusion of teeth to avoid lateral pressure.

**Figure 8:**
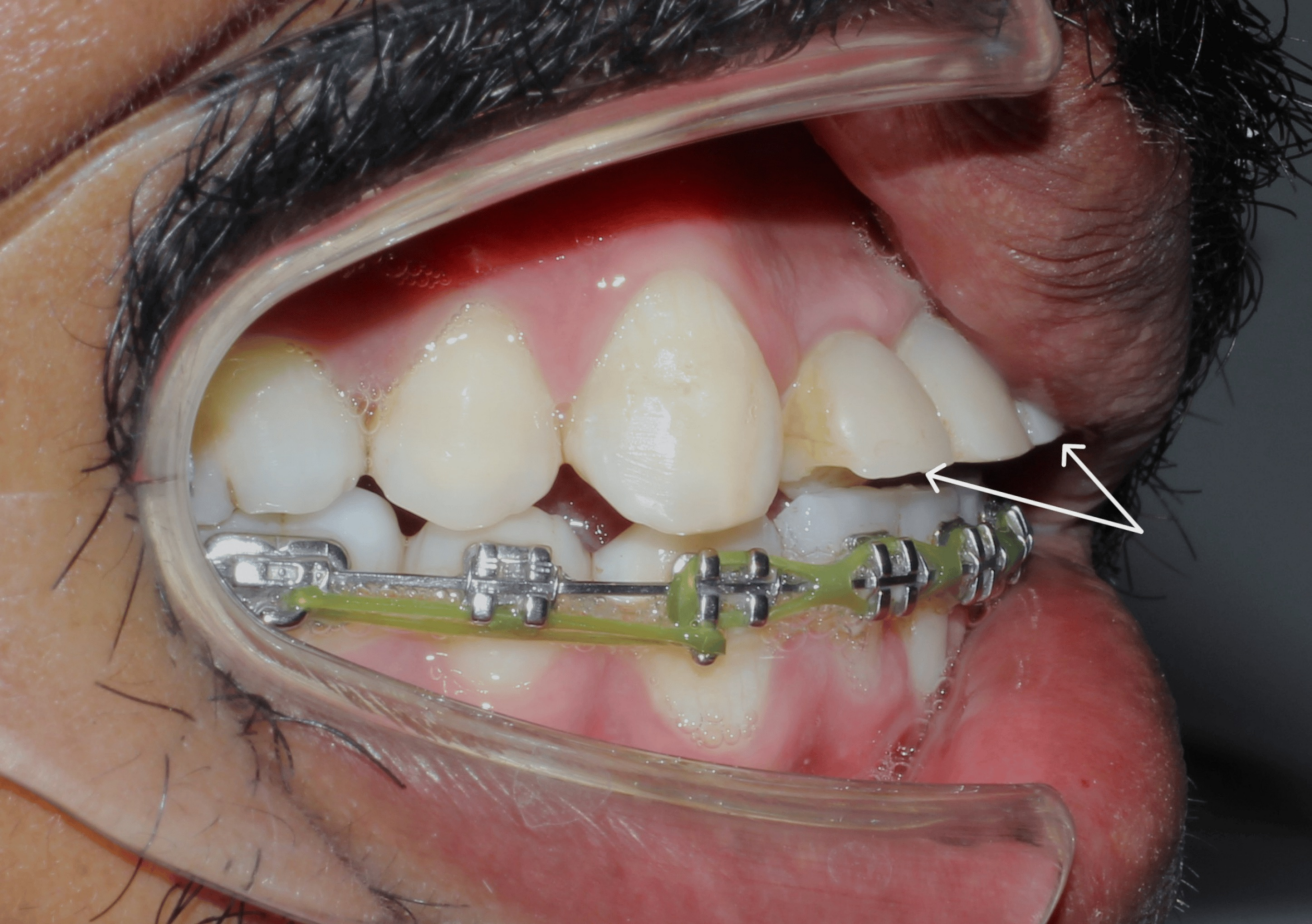
Lateral view showing temporisation of teeth with disclusion of teeth.

The temporary bridge was fabricated with direct technique by using Protemp (3M, Saint Paul, MN, USA) (Figure 7 and Figure 8).

**Figure 9:**
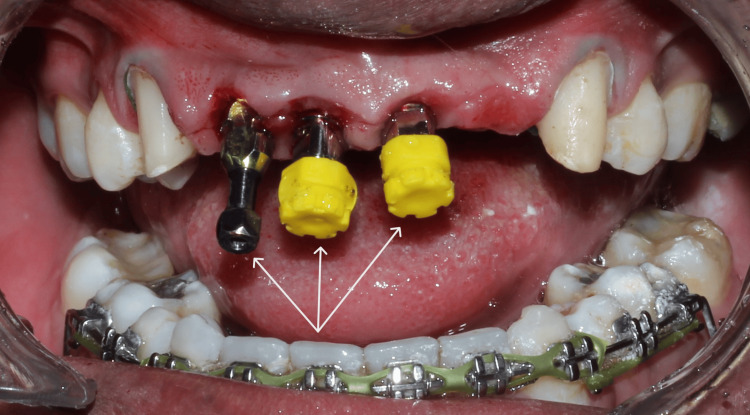
Impression post placed after satisfactory healing of the papillary mucosa over the implant site for a pick-up and transfer combination of impression making.

**Figure 10:**
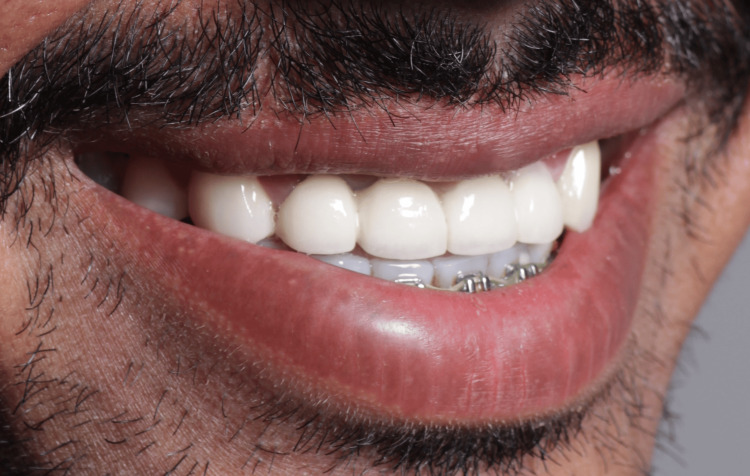
Final fixed anterior bridge delivered.

**Figure 11:**
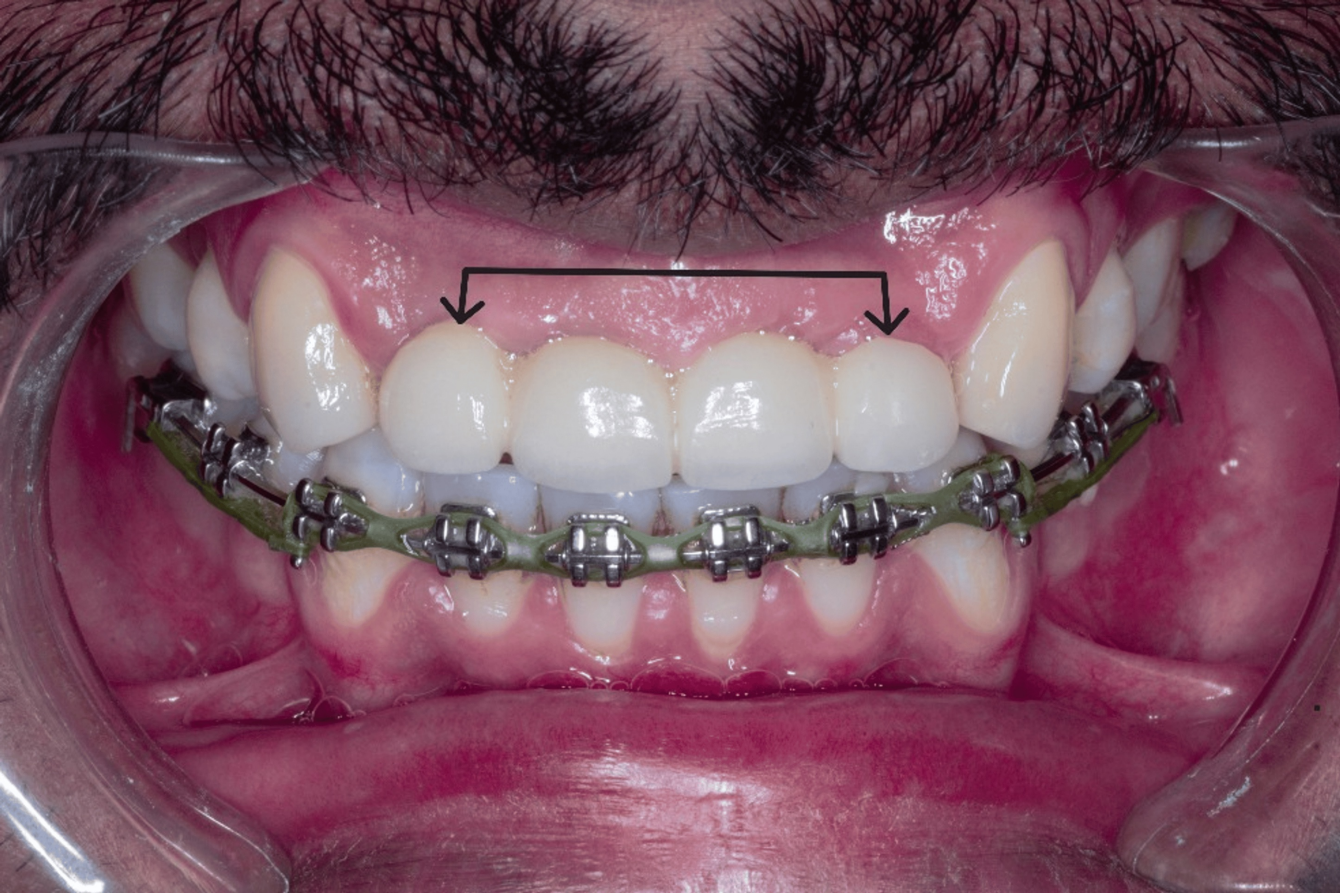
Upper anterior bridge profile in the esthetic zone.

**Figure 12:**
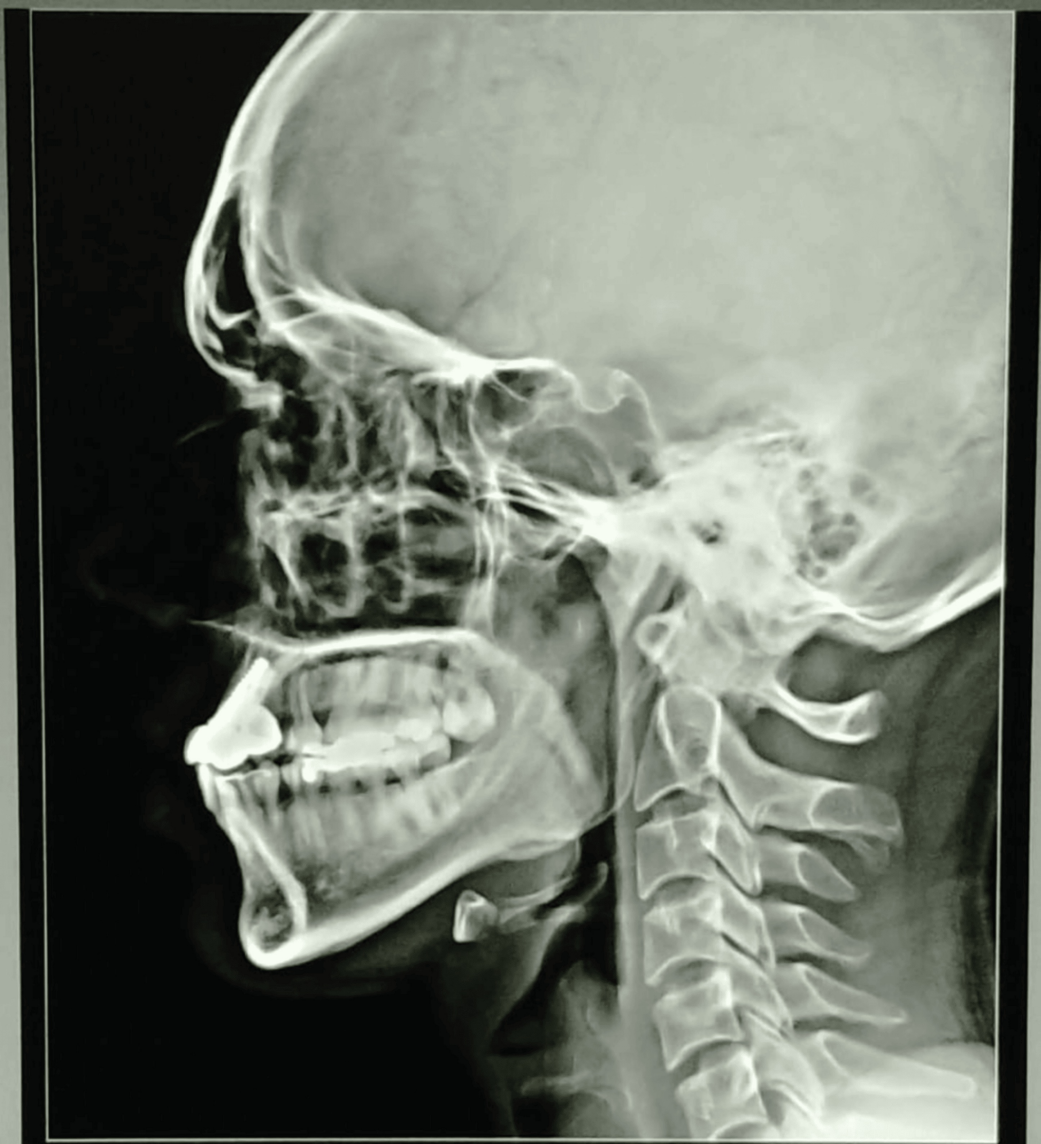
Post-op lateral cephalogram.

Following the implant insertion procedure, the patient underwent routine examinations at two, four, and seven weeks as well as at three and four months. No complaints of pain or harmful effects were reported. During the post-operative follow-up visits, the gingival margin around the implant provisional crown appeared stable, with functional attachment. The orthodontic treatment for the realignment of lower teeth continued for a period of one year, following which the prosthetic phase for permanent prosthesis was initiated. Abutments with pick-up impression cap were placed in Myriad implants with respect to 11 and 21, and impression post in Bioline implant with respect to 12 was placed in their respective position (Figure 9). Porcelain fused to metal bridge fabricated with direct metal laser sintering technology was cemented as a definitive prosthesis which then replaced the temporary bridge (Figure 10 and Figure 11). After receiving the final crown restoration, the patient was monitored for a year. Following implant loading, recall appointments were scheduled every six months. The implant held its stability and performed as intended during these recall visits. The peri-implant gingiva was pink and healthy, with no bleeding on probing. A periapical radiograph was taken to assess the marginal bone level on the implant's mesial and distal sides. In contrast to the radiograph obtained during the final crown delivery, this one showed a stable marginal bone level surrounding the implant with no signs of peri-implant radiolucency (Figure 12). During the one-year follow-up period, a satisfactory cosmetic outcome was noted by immediate implant placement and provisionalization along with bone grafting.

**Figure 13:**
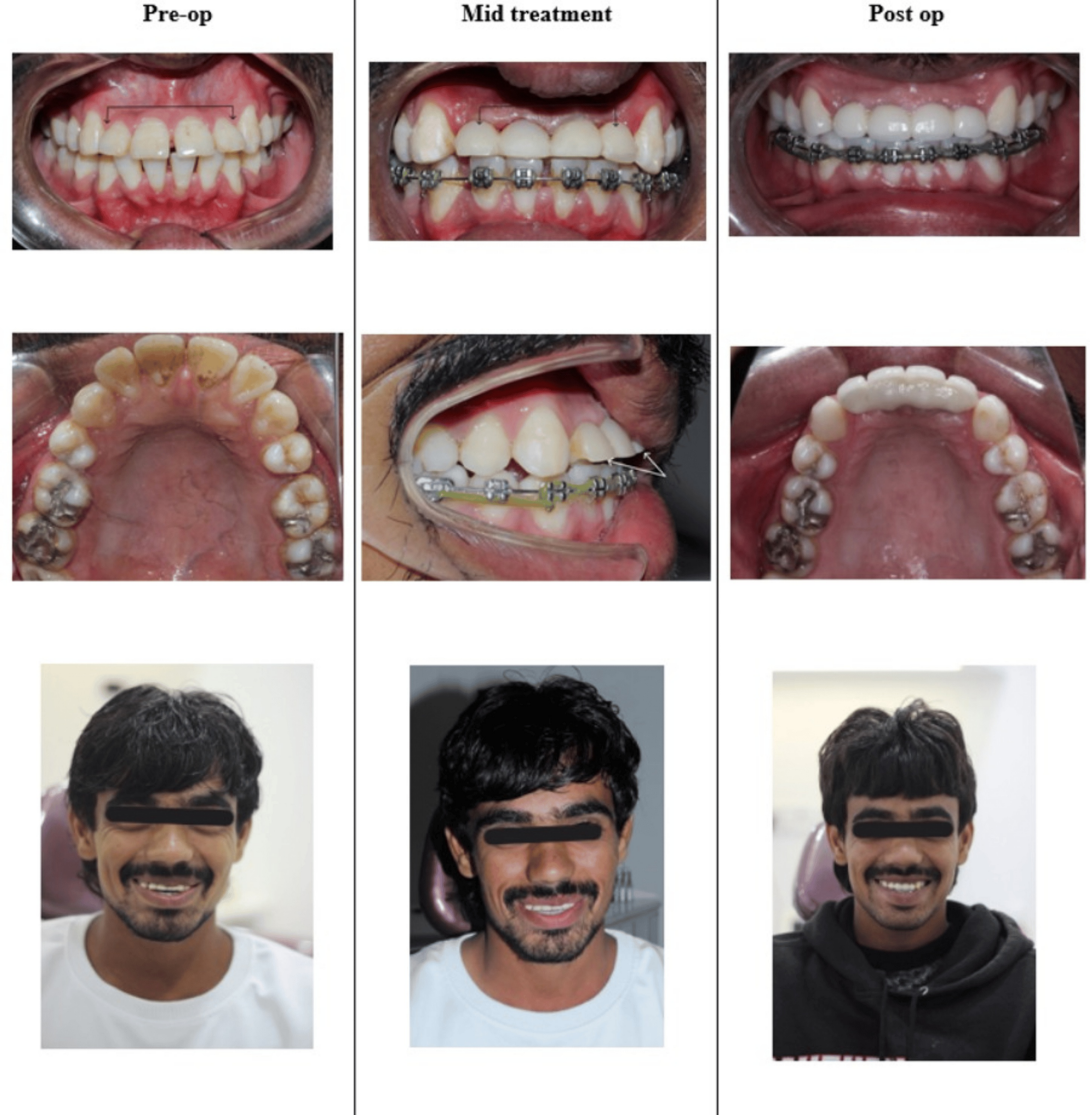
Comparison of pre-operative, mid-treatment, and post-operative photographs.

Figure 13 depicts a comparison of pre-operative, mid-treatment, and post-operative photographs.

## Discussion

Immediate implant placement refers to the placing of a dental implant on the same day to up to 10 days from the day of tooth extraction in a single surgical procedure [7]. Wöhrle's procedure for immediate implant placement and provisionalization in the esthetic zone has been shown to have great success and survival rates while maintaining stable gingival architecture [8]. However, studies have generally concentrated on success and survival rates, as well as radiographic marginal bone levels, with little emphasis on soft tissue factors like proximal papilla and facial gingival levels [9].

A classification system for extraction sockets in the maxillary anterior region, introduced in 2008, outlines specific criteria for both soft and hard tissue components to guide successful immediate implant placement. In this case, the extraction socket falls under grade I, making it suitable for immediate implant placement. However, careful case selection is crucial due to the challenges posed by immediate implant placement and provisionalization.

The implant was strategically positioned in the center of the final restoration, with a minimum clearance of 1.5 mm between the implant fixture and adjacent natural teeth. A recent study highlighted the significance of a buccal bone wall thickness of at least 1.5 mm in accounting for post-implant placement alterations and the possibility of peri-implantitis [10].

To improve the emergence profile, the implant platform was placed apicocoronally, at least 3 mm from the cementoenamel junction of the next tooth [11]. Kan et al.'s [12] classification suggests that the palatal aspect has a substantial amount of bone and is a good candidate for rapid implant placement. Studies in the fields of clinical and histology have shown that ridge dimension varies significantly after tooth extraction. Procedures for bone augmentation have shown success in increasing bone fill and correcting deficiencies at the locations of immediate implants [13].

It is possible to preserve a desirable hard tissue contour in both vertical and horizontal planes when bone grafting materials are used to bridge the implant-socket gap, according to both clinical and histologic studies [14]. In this case, freeze-dried bone allograft proved effective in maintaining esthetically pleasing hard tissue contours with minimal changes in marginal bone levels over an extended period.

Chu et al. proposed the dual-zone concept, which involves the use of bone grafts in both the bone and tissue zones to preserve volume in both hard and soft tissues. In their case, bone grafts have been placed in the space between the implant fixture and the buccal plate, but not to the soft tissue level [15]. The present case uses immediate placement and loading along with augmentation with a mixture of xenografts and autogenous bone harvested from the osteotomy site. There has been evidence that the addition of xenograft bone graft particles to prevent ridge collapse during the insertion of implants in fresh extraction sockets with the placement of immediate interim restoration reveals stable results in both hard and soft tissues [16].

There has been data which shows no statistical difference in the success of the immediate loading protocol as opposed to the conventional loading protocol [17]. Contributing to the success of immediate loading, there has been documented evidence of long-term success up to 10 years [18].

Achieving stable soft and hard tissue outcomes in esthetic implant therapy is challenging. Implant placement is important; implants positioned buccally are linked to increased mid-buccal mucosal recession. Using a flapless approach during immediate implant placement and instant provisionalization has been shown to reduce midfacial mucosal recession compared to open-flap placement and delayed restoration, respectively [19].

Overall, careful consideration of anatomical factors, proper surgical techniques, and appropriate timing of procedures contribute to minimizing midfacial mucosal recession and achieving successful outcomes in esthetic implant therapy [20]. Achieving primary stability of the implant is crucial for immediate implant provisionalization, with a torque greater than 32 Ncm necessary for success. In this case, primary stability was achieved with an insertion torque exceeding 35 Ncm.

## Conclusions

The objective of this case report was to examine the surgical steps involved in immediate implant placement and provisionalization following tooth extraction. Success relies heavily on careful planning and meticulous case selection. Immediate implant placement and provisionalization preserve the natural architecture of both soft and hard tissues, leading to predictable and excellent esthetic results. Long-term success is contingent upon achieving primary stability during implant placement and careful provisionalization design to avoid any contact during the healing phase. The described technique includes a flapless extraction, immediate implant placement, and provisionalization without functional fatigue during healing, making it a viable choice for single-tooth replacement in the esthetic zone.
